# Volume Management in the Critically Ill Patient with Acute Kidney Injury

**DOI:** 10.1155/2013/792830

**Published:** 2013-02-07

**Authors:** Mary Labib, Raeesa Khalid, Akram Khan, Supriya Khan

**Affiliations:** ^1^Nephrology & Hypertension, Oregon Health and Science University, 3181 SW Sam Jackson Park Road, CH12R, Portland, OR 97239-3098, USA; ^2^Pulmonary & Critical Care Medicine, Oregon Health & Science University, 3181 SW Sam Jackson Park Road, UHN67, Portland, OR 97239-3098, USA

## Abstract

Acute kidney injury (AKI) frequently occurs in the setting of critical illness and its management poses a challenge for the intensivist. Optimal management of volume status is critical in the setting of AKI in the ICU patient. The use of urine sodium, the fractional excretion of sodium (FeNa), and the fractional excretion of urea (FeUrea) are common clinical tools used to help guide fluid management especially further volume expansion but should be used in the context of the patient's overall clinical scenario as they are not completely sensitive or specific for the finding of volume depletion and can be misleading. In the case of oliguric or anuric AKI, diuretics are often utilized to increase the urine output although current evidence suggests that they are best reserved for the treatment of volume overload and hyperkalemia in patients who are likely to respond to them. Management of volume overload in ICU patients with AKI is especially important as volume overload has several negative effects on organ function and overall morbidity and mortality.

## 1. Introduction

Acute kidney injury (AKI) is a frequent complication in critically ill patients in the intensive care unit (ICU) with an incidence ranging from 17.5% to 78% [1–5]. Management of volume status in critically ill patients with AKI is difficult as it is often accompanied by oliguria or anuria as well as total body fluid overload and tissue edema. AKI increases the risk of mortality and often occurs in the setting of sepsis or other forms of shock [6]. While the early goal-directed therapy study showed the benefit of adequate volume repletion in critically ill patients with septic shock [7], there are detrimental effects associated with salt and water overload which can result from resuscitation with crystalloids or colloids. These include worsening of lung function and difficulty of wound healing [8, 9].

In this paper we will focus on the role of intravenous fluids (IVFs) and diuretics for the management of volume status in critically ill patients with AKI. We will also discuss the differences between oliguric and nonoliguric renal failure and the effects on outcomes of “converting” patients from oliguric to nonoliguric renal failure.

## 2. Epidemiology and Mortality of AKI in the ICU

The Acute Dialysis Quality Initiative (ADQI) published the Risk, Injury, Failure, Loss, End-Stage Kidney Disease (RIFLE) definitions for AKI in 2004 [10], developing a consensus definition of AKI that could be used in studies rather than the 30 plus definitions that had been used in previous studies. This was revised by the Acute Kidney Injury Network (AKIN) in 2007 [11] (Table 1). Bagshaw et al. showed that there was no advantage of using one criterion over the other and that the sensitivity, robustness, and predictive ability using both definitions to classify AKI within the first 24 hours of admission into the ICU were similar [6].

Regardless of the classification system used, the incidence of AKI in patients admitted to the ICU is high, ranging from 18 to 78% [1–6]. For example, the incidence of AKI in patients undergoing cardiothoracic surgery in one study varied between 18.9% and 26.3% depending upon if the RIFLE or AKIN criteria were used [1]. In-hospital AKI is a significant risk factor for in-hospital mortality. In one study, the odds ratio for in-hospital mortality was 3.29 in patients with AKI versus those without AKI [6]. In multiple other studies, AKI of any RIFLE class or AKIN stage significantly increases mortality, with a mortality rate in studies ranging from approximately 8% to 72% [4, 5]; oliguric AKI carries a higher mortality than nonoliguric AKI [12]. It is unclear if the reason for the increased mortality in oliguric and anuric AKI is the underlying increased severity of the renal injury due to increased severity of illness or if there is something else inherent to AKI that increases mortality.

## 3. Administration of Intravenous Fluids (IVF) in AKI

Given the high mortality of in-hospital AKI and the high incidence of AKI especially in the ICU, it is important to consider the factors that can affect its management, including volume status. Maintaining adequate intravascular volume is an important part of the therapy of septic shock as demonstrated by Rivers et al. [7]. Guaranteeing adequate renal perfusion and intravascular volume is also important in the prevention and therapy of AKI in the ICU [8]. Clinicians in the ICU have traditionally used urinary chemistries such as the urine sodium and fractional excretion of sodium (FeNa) and urea (FeUrea) to help differentiate between pre-renal and intrarenal causes of AKI and to help guide further administration of IVF [13]. There are several circumstances, however, in which these traditional urinary biomarkers can be misleading, including in sepsis [14, 15], myoglobinuria [16], contrast-induced nephropathy [17], acute glomerulonephritis [18], cirrhosis [18], congestive heart failure [18], and use of calcinerin inhibitors (CNIs) [19] or diuretics [20]. A low FeNa, or low urine sodium reflects poor renal perfusion of any cause, not exclusively volume depletion as an etiology of AKI, and there are many causes of “low FeNa ATN” as listed above (Table 2). FeUrea has been felt to perhaps be a better test to evaluate prerenal AKI in the setting of diuretic administration; however, the sensitivity and specificity of the FeUrea was poor in a multicenter cohort study [21]. Given the problems with interpretation of the urine sodium, FeNa, and similar urinary chemistries, it is important to use them as just one important piece of data within the context of the patient's overall clinical scenario rather than giving additional volume on the strength of a low FeNa alone; important adjuncts to the urine chemistries include physical examination for signs of volume depletion or overload and use of invasive hemodynamic monitoring as done in recent trials looking at fluid management in acute lung injury [22–24].

## 4. Administration of Diuretics in AKI and Conversion of Oliguric to Nonoliguric Renal Failure

Oliguria and anuria are common problems facing ICU clinicians caring for patients with AKI. As above, studies in the past have suggested that oliguric AKI carries a higher mortality than nonoliguric AKI [12]. There is some physiologic sense to the idea that maintaining urinary flow during AKI may be beneficial. A few studies have shown that inhibition of the sodium/potassium chloride cotransporter (NKCC2) by loop diuretics reduces active sodium transport, decreases oxygen consumption, and may decrease potential ischemic injury [25]. In light of this, many ICU clinicians have tried to convert patients from oliguric to nonoliguric AKI with the use of diuretics, typically loop diuretics [26]. Unfortunately, these theoretical benefits of increasing the urinary flow have not translated into similar improvement in the clinical outcome in several studies (Table 3) [8, 9, 12, 25, 27]. It has been suggested that patients with nonoliguric AKI either de novo or in response to diuretics may have had better outcomes than those with oliguric AKI due to a lower level of severity of their primary etiology of AKI compared to the oliguric patients. It is also possible that poor outcomes may have been due to a delay in nephrology consultation and/or provision of renal replacement therapy while clinicians were waiting for a response from the oliguric patients. In these studies while diuretics did not improve clinical outcomes, they were effective in improving the urine output in those patients who could respond to them. This suggests that diuretics should be used in patients with AKI in the ICU for the management of the volume overload, rather than just for converting patients from oliguric to nonoliguric AKI. In addition, loop diuretics promote kaliuresis and can be used for the management of hyperkalemia in patients who are not oliguric or anuric and more likely respond to diuretic therapy.

## 5. Avoidance of Volume Overload in ICU Patients with AKI

A frequent complication of oliguria and anuria in ICU patients with AKI is the development of volume overload. As previously noted, volume administration in patients with AKI should be done cautiously and with close attention paid to the patient's overall clinical status including hemodynamics, intravascular volume, and respiratory status. Diuretics can be given to patients with AKI to combat volume excess as this can have negative effects on multiple organ systems including heart, skin, kidneys, and lungs [9, 33–36].

In patients with established AKI unresponsive to fluid administration, fluid restriction is the treatment of choice. Patients undergoing any major surgery typically gain 3–6 kg due to fluid administration which has been associated with worse cardiopulmonary and surgical wound healing outcomes and overall outcomes [9]. Brandstrup et al. performed a randomized assessor blinded multicenter study looking at perioperative fluid management strategies in patients undergoing colorectal surgery [9]. They found that a restricted intravenous fluid regimen significantly reduced postoperative cardiopulmonary complications and tissue/wound healing complications in intention to treat and per protocol analyses. They did not note any harmful adverse effects of the fluid restrictive strategy. This suggests that fluid restriction in surgical patients with AKI may lead to improved wound healing and overall outcomes. 

The kidney is an encapsulated organ and fluid congestion and elevated venous pressures can lead to a decrease in renal blood flow and glomerular filtration rate (GFR) [34]. Fluid overload in critically ill patients, especially those in the surgical and trauma ICUs, may also predispose them to the development of intra-abdominal hypertension (IAH) which is an additional risk factor for the development of AKI [34]. 

Fluid overload is also associated with a greater incidence of nonrecovery of renal function in patients starting renal replacement therapy [35]. Heung et al. performed a retrospective, single-center analysis of 170 patients who started renal replacement therapy (RRT) for AKI attributed to acute tubular necrosis (ATN) [35]. They found that those patients with fluid overload had a worse rate of renal recovery at one year than those euvolemic at the start of renal replacement therapy.

 The Fluid and Catheter Treatment Trial (FACTT), a multicenter, randomized controlled trial evaluating a conservative versus liberal fluid management strategy for 1000 patients with acute lung injury, did not show a mortality benefit, but did show a benefit in ventilator free days in patients randomized to a fluid conservative strategy [24]. A subgroup analysis of patients who developed AKI in this study was done looking at the association of postrenal injury fluid balance and diuretic use with 60-day mortality [23]. Of the 1000 patients studied in the parent FACTT trial, 306 patients developed renal injury. Post-AKI fluid balance was significantly associated with the risk of death in this subgroup with the risk of death approximately 1.6-fold higher per liter/day of fluid accumulated. Diuretic use was associated with decreased mortality at 60 days, except when adjusted for fluid balance. It is possible that patients who received diuretics had an improvement in mortality because they achieved better fluid balance with decreased accumulation of fluid. 

## 6. Recommended Strategy for Volume Management

Optimal management of volume status in AKI requires close collaboration between the critical care physician, nephrologist, and other subspecialists participating in the care of the patient. Intravenous fluids play a critical role in the resuscitation of the critically ill patient with sepsis [7, 37], but the overzealous fluid resuscitation has been associated with increased complications, increased length of ICU and hospital stay, and increased mortality [8, 9, 38]. In the opinion of the authors of this paper, an accurate assessment of body weight, fluid balance and fluid responsiveness (an increase in stroke volume of 10–15% after the patient receives 500 ml of crystalloid over 10–15 minutes) is central to the optimization of volume management in patients with AKI [39]. This would be dependent upon the resources of the treating institution and could range from clinical indices of adequate perfusion such as mean arterial pressure, urine output, capillary refill and central venous pressure monitoring, laboratory data such as serum lactate, arterial blood gas, and central or mixed venous oxygen saturation, or more advanced tools to determine fluid responsiveness as defined by fluid responsiveness to passive leg raising with or without bioreactance, pulse pressure or stroke volume variation, or transpulmonary thermodilution with global end-diastolic volume/extravascular lung water measurement [38–41]. 

The authors of this paper would recommend an approach that incorporates adequate resuscitation with intravenous fluids and vasopressors assessing for volume status and fluid responsiveness based upon the resources of the treating facility. In addition, in individuals who develop AKI, in addition to other measures, urine chemistries may be helpful in determining the need for intravenous fluid administration with the caveats addressed previously; a low FeNa or FeUrea may not equal volume depletion and may be just an indicator of inadequate renal perfusion. Hypovolemic patients will benefit from intravenous fluid while fluid responsive. If the patient is volume replete and oliguric, we would advise a strategy of fluid restriction to avoid further fluid overload. Diuretics may be considered to address fluid overload and mild hyperkalemia. There may be no benefit to the use of diuretics for the treatment of oliguria, but there is some benefit of inducing a net negative volume status if possible with diuretics. If the patient remains oliguric and volume overloaded despite an adequate trial of high-dose intravenous diuretics, we would recommend considering nephrology consultation and RRT.

## 7. Conclusion

Adequate resuscitation is the key to the therapy of sepsis and other forms of shock commonly encountered in the ICU. However, overadministration of fluids can have negative effects on multiple organ systems and fluid overload is associated with increased mortality. Fluid overload is both a consequence and a risk factor for the development of AKI in critically ill patients. AKI is a frequent complication of ICU stays and is associated with an increased mortality. AKI further complicates the fluid management in these patients who have limited ability to deal with salt and water excess due to decreased GFR and urine output. Oliguric renal failure may be associated with a higher risk of death than nonoliguric renal failure in part because of the diminished ability to manage volume excess, but also because oliguric renal failure may reflect worse renal injury and an overall sicker patient. 

Making an accurate assessment of intravascular volume status and volume responsiveness using the tools available at the treating institution is paramount in the critically ill patient with AKI. The authors would recommend an approach that incorporates ensuring adequate intravascular volume, while avoiding the administration of intravenous fluids when the patient is no longer volume responsive to avoid the volume overload which can be associated with adverse outcomes. In patients who are fluid overloaded and oliguric, diuretics can be helpful in some cases. While there is no evidence that converting patients from oliguric to nonoliguric renal failure has a beneficial effect on mortality or recovery of renal function after AKI, avoiding fluid overload and inducing a net negative fluid balance correlate with better outcomes after AKI. For this reason, the use of diuretics in critically ill patients with AKI should be limited to management of volume overload and in some cases hyperkalemia, when the patient is making urine. A failure of diuretics to increase urine output in a critically ill patient should be an indication that nephrology consultation may be needed and that initiation of RRT may need to be considered.

## Figures and Tables

**Table 1:** Comparison of RIFLE and AKIN criteria for the acute kidney injury (AKI).

RIFLE category	Serum creatinine criteria	Urine output criteria

The Acute Dialysis Quality Initiative (ADQI) criteria for the definition and classification of AKI (i.e., RIFLE criteria)		
Risk	Increase in serum creatinine ≥1.5 X baseline or decrease in GFR ≥25%	<0.5 mL/kg/h for ≥6 h
Injury	Increase in serum creatinine ≥2.0 X baseline or decrease in GFR ≥50%	<0.5 mL/kg/h for ≥12 h
Failure	Increase in serum creatinine ≥3.0 X baseline or decrease in GFR ≥75% or an	<0.3 mL/kg/h ≥24 h or
	absolute serum creatinine ≥4.0 mg/dL with an acute rise of at least 0.5 mg dL	anuria ≥12 h
Loss	Persistent acute renal failure—complete loss of kidney function ≥4 weeks	N/A
EskD	End-stage kidney disease ≥3 months	N/A

AKIN stages	Serum creatinine criteria	Urine output criteria

The proposed Acute Kidney Injury Network (AKIN) criteria for the definition and classification of AKI		
Stage 1	Increase in serum creatinine ≥0.3 mg/dL or increase to ≥150–199% (1.5- to 1.9-fold) from baseline	<0.5 mL/kg/h for ≥6 h
Stage 2	Increase in serum creatinine to 200–299% (>2–2.9-fold) from baseline	<0.5 mL/kg/h for ≥12 h
Stage 3	Increase in serum creatinine to ≥300% (≥3-fold) from baseline or serum creatinine	<0.3 mL/kg/h ≥24 h or
	≥4.0 mg/dL with an acute rise of at least 0.5 mg/dL or initiation of RRT	anuria ≥12 h

**Table 2:** Clinical scenarios in which the urine sodium and FeNa may be unreliable.

Sepsis
Congestive heart failure
Myoglobinuria and hemoglobinuria
Contrast nephropathy
Cirrhosis
Acute glomerulonephritis
Use of calcineurin inhibitors
Use of diuretics

**Table 3:** Studies examining the effects of diuretics in AKI.

Reference	Study type	Population	*n *	Effect of diuretics
Mehta et al. (2002) [27]	Retrospective cohort	Patients in 4 teaching hospital ICUs affiliated with the University of California with nephrology consultations, medical and surgical ICU patients	552	Increased risk of death or nonrecovery of renal function (OR 1.77), magnified when patients who died within the first week after consultation were excluded (OR 3.12)

Uchino et al. (2004) [28]	Prospective multicenter, epidemiological study	ICU patients with the following etiologies of AKI: severe sepsis/septic shock (43.8%), major surgery (39.1%), low cardiac output 29.7%), hypovolemia (28.2%)	1734	No statistically significant difference in groups with or without diuretic use

Shiliday et al. (1997) [29]	Prospective, randomized, double-blind placebo-controlled trial	ICU patients at a single center	92	Increase in urine output with diuretics Improvement in mortality for those who became nonoliguric but had lower APACHE II scores at baseline. No difference in mortality between those who became nonoliguric with placebo versus diuretics

Cantarovich et al. (2004) [30]	Prospective, randomized, double-blind, placebo-controlled trial	Multicenter trial, 13 ICUs, 10 nephrology wards	338	Increase in urine output with diuretics No improvement in survival, renal recovery, number of dialysis sessions, or duration of need for dialysis between the two groups

Van der Voort et al. (2009) [31]	Prospective, randomized, double-blind, placebo-controlled trial	ICU patients at a single center treated with CVVH	72	Increase in urine output with diuretics. No improvement in duration of renal failure or rate of renal recovery

Wu et al. (2012) [32]	Prospective, multicenter, observational study	Postsurgical ICU patients receiving hemodialysis	572	Higher doses of diuretics were associated with hypotension and increased mortality
